# A Web-Based Stratified Stepped Care Platform for Mental Well-being (TourHeart+): User-Centered Research and Design

**DOI:** 10.2196/38504

**Published:** 2023-03-22

**Authors:** Winnie W S Mak, Sin Man Ng, Florence H T Leung

**Affiliations:** 1 Department of Psychology The Chinese University of Hong Kong Shatin Hong Kong

**Keywords:** user-centered design, qualitative research, usability testing, mental health, self-care, stratified stepped care, internet-based psychological interventions

## Abstract

**Background:**

Internet-based mental health interventions have been demonstrated to be effective in alleviating psychological distress and promoting mental well-being. However, real-world uptake and engagement of such interventions have been low. Rather than being stand-alone interventions, situating internet-based interventions under a stratified stepped care system can support users to continue with mental health practice and monitor their mental health status for timely services that are commensurate with their needs. A user-centered approach should be used in the development of such web-based platforms to understand the facilitators and barriers in user engagement to enhance platform uptake, usability, and adherence so it can support the users’ continued adoption and practice of self-care for their mental health.

**Objective:**

The aim of this study was to describe the design process taken to develop a web-based stratified stepped care mental health platform, TourHeart+, using a user-centered approach that gathers target users’ perceptions on mental self-care and feedback on the platform design and incorporates them into the design.

**Methods:**

The process involved a design workshop with the interdisciplinary development team, user interviews, and 2 usability testing sessions on the flow of registration and mental health assessment and the web-based self-help interventions of the platform. The data collected were summarized as descriptive statistics if appropriate and insights are extracted inductively. Qualitative data were extracted using a thematic coding approach.

**Results:**

In the design workshop, the team generated empathy maps and point-of-view statements related to the possible mental health needs of target users. Four user personas and related processes in the mental health self-care journey were developed based on user interviews. Design considerations were derived based on the insights drawn from the personas and mental health self-care journey. Survey results from 104 users during usability testing showed that the overall experience during registration and mental health assessment was friendly, and they felt cared for, although no statistically significant differences on preference ratings were found between using a web-based questionnaire tool and through an interactive chatbot, except that chatbot format was deemed more interesting. Facilitators of and barriers to registering the platform and completing the mental health assessment were identified through user feedback during simulation with mock-ups. In the usability testing for guided self-help interventions, users expressed pain points in course adherence, and corresponding amendments were made in the flow and design of the web-based courses.

**Conclusions:**

The design process and findings presented in the study are important in developing a user-centric platform to optimize users’ acceptance and usability of a web-based stratified stepped care platform with guided self-help interventions for mental well-being. Accounting for users’ perceptions and needs toward mental health self-care and their experiences in the design process can enhance the usability of an evidence-based mental health platform on the web.

## Introduction

### Proliferation of Internet-Based Mental Health Interventions

Using the internet has become a daily routine among the majority around the world. In Hong Kong, about 92% of households had internet access and more than 92% of people aged ≥10 years had used the internet in the past year based on figures in 2020 [1]. The universality of internet access has escalated the development of e–mental health [2-5]. Mounting evidence in support of the use of internet-based interventions for alleviating psychological distress has also been published [6-11]. Nevertheless, users generally still prefer face-to-face interventions over web-based interventions [12,13] and their adherence to web-based interventions was generally low [14,15]. Thus, improving the implementation of internet-based interventions through user-centered design could potentially increase uptake of and adherence to web-based services, which can offset the current imbalance between demand and supply for mental health services [16,17]. Across many regions of the world, help-seeking for professional mental health services tends to be low because of stigmatization, high costs of services in the private sector, and long waiting time for public services [18-20]. Through accessing web-based services, evidence-based services can be made immediately available to users anytime, anywhere. Thus, internet-based interventions may provide alternatives for a sizable proportion of people who are in need and would not otherwise seek psychological interventions in person. However, many of these web-based self-help resources either lack rigorous research evidence, or their ratings and popularity do not correspond to the extent of research evidence on their functions and features [21-25].

### From TourHeart to TourHeart+

To address these challenges, the first author and her team developed a web-based platform (TourHeart) based on a stratified stepped care model with evidence-based psychological interventions for mental health promotion, illness prevention, and treatment of common mental disorders that have been developed locally in Hong Kong [26-34]. By applying the least restrictive and self-correcting principles, this approach enables users to step up or down on the intensity of mental health services based on their mental health status [28]. According to the qualitative evaluation conducted with users and other stakeholders of TourHeart [30], suggestions for revamping the platform included developing a chatbot that could provide prompt and anonymous support, informing users of available options, increasing transparency of the platform, and focusing on personal recovery.

A completely new web-based platform (TourHeart+) was developed to address users’ feedback and evolving mental health needs. Although TourHeart+ continued to use the stratified stepped care approach, unlike most other stepped care systems that are symptom-based and professionally driven, the design of TourHeart+ emphasized users’ input and preferences. Moreover, based on the 2 continua model of mental health [35,36], the TourHeart+ platform targets working adults in Hong Kong who would like to take care of their mental well-being regardless of absence or presence of psychological distress. To develop the platform using user-centered design, a design workshop, user interviews, and user experience (UX) usability testing were conducted to identify relevant concerns and preferences of the users, as well as determine format, design, and contents of the platform [37]. This paper documents the multistep design process used in understanding target users’ needs and preferences and in developing the registration flow, mental health assessment, and web-based courses, which are the core features of TourHeart+.

## Methods

### Study Design

The aim of the series of research activities conducted was to ensure the platform design was informed by and met users’ needs and preferences. A user-centered approach was taken to (1) empathize with users to derive design considerations and (2) test prototypes with target users to improve the design on usability. Refer to Tables 1 and 2 on the 2-phase process’s objectives and outcomes. Phase 1 focused on defining design considerations by empathizing with users through a design workshop with the development team and interviews with target users. Phase 2 focused on usability testing on the onboarding flow for registration and initial assessment of mental health status and web-based self-help courses.

**Table 1:** Research objectives and outcomes–phase 1.^a^

Research activity	Design workshop	User interviews
Objectives	Allow stakeholders to empathize and share knowledge about target users and to define problem statements for the design	Understand target users’ values, motivations, and experiences on self-care for mental health
Outcomes	References for user interviews empathy map of 2 user groups problem statements for each user groups	User personas User journey Design considerations for TourHeart+ platform

^a^Define design considerations by empathizing with users.

**Table 2:** Research objectives and outcomes–phase 2.^a^

Research activity	Usability testing on the onboarding flow including registration and mental health assessment	Usability testing on the web-based self-help courses
Objectives	Develop an inviting and smooth onboarding flow to engage and retain users	Ensure design of courses and exercises can facilitate users’ learning and maintain adherence
Outcomes	General impressions on the platform Pain points and opportunities Decision on delivery format of mental health assessment	Frictions in understanding and choosing a course to start Frictions in using course and exercise contents

^a^Improve platform usability by testing prototypes with target users.

### Phase 1: Define Design Considerations by Empathizing With Users

#### Design Workshop

**Figure 1:**
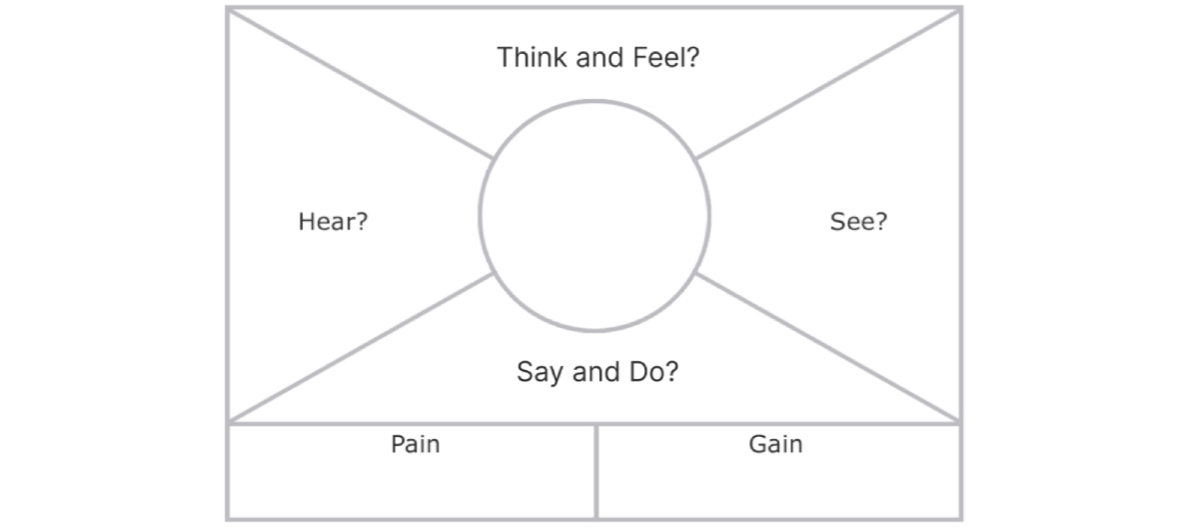
Example of empathy map.

**Figure 2:**
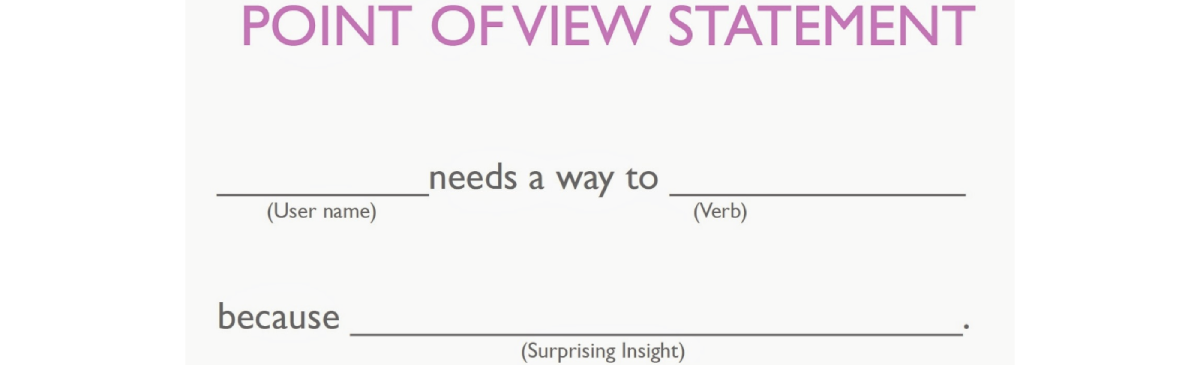
Example of point-of-view statement.

A workshop was conducted with the entire development team with members having a background in psychology, engineering, journalism and communication, and business administration to generate collaborative knowledge by shifting the perspective from “designers” to “users.” The workshop consisted of 2 key activities. First, the team empathized with the 2 user segments, 1 with none to mild levels of distress and another with moderate to severe levels of distress, by using an empathy map (Figure 1). An empathy map is a visualization tool that helps the team to identify target users’ thoughts, feelings, motivations, desires and needs. Second, based on the map created and discussions among the team, the team created problem statements using “point-of-view statement” framework (Figure 2). This exercise intends to break down the possible mental health issues that the TourHeart+ platform will meet and the possible approaches to tackle these issues.

The workshop was held on March 10, 2021, through a web-based collaborative whiteboard platform, Miro, and was led by the UX researcher in the team. The UX researcher gained insights from the workshop and used them as references for subsequent research activities.

#### User Interviews

Interviews with target users were semistructured and followed a set of interview questions created by the UX researcher and research assistant in the team. The aim of the interviews was to gain an in-depth understanding of target users on mental health self-care. The research questions included (1) *what are their perceptions toward mental health;* (2) *how do they make sense of their own mental health status;* and (3) *What are their experiences, attitudes, goals, and behaviors toward mental health self-care in relation to the TourHeart+ platform?*

The user interviews were conducted between April 15, 2021, and April 28, 2021. Eligible participants, who were aged between 18 and 60 years and in the work force, were contacted by research staff to an arrangement for a 1-hour interview. The intention of the user interviews was to come up with users’ personas and journeys to facilitate us in designing a platform that can address their needs and preferences.

### Phase 2: Improve Platform Usability by Testing Prototypes With Target Users

#### Usability Testing on the Onboarding Flow Including Registration and Mental Health Assessment

To attract users to join the platform, it is important to ensure that the onboarding flow is smooth and inviting. Hence, the purpose of this usability test was to discover areas for improvement for the initial process that can be addressed before the platform launch. Usability testing was conducted through an unmoderated user test to maximize the sample size and collect relevant feedback quickly [38]. The testing included a prototype of the flow, followed by a survey to obtain participants’ feedback. Three key research questions were set for the study: (1) What are the users’ general impressions toward the platform after completing the registration and mental health assessment? (2) What are the pain points and opportunities to improve the flow? (3) Should we adapt a typeform format or chatbot format for mental health assessment?

**Figure 3:**
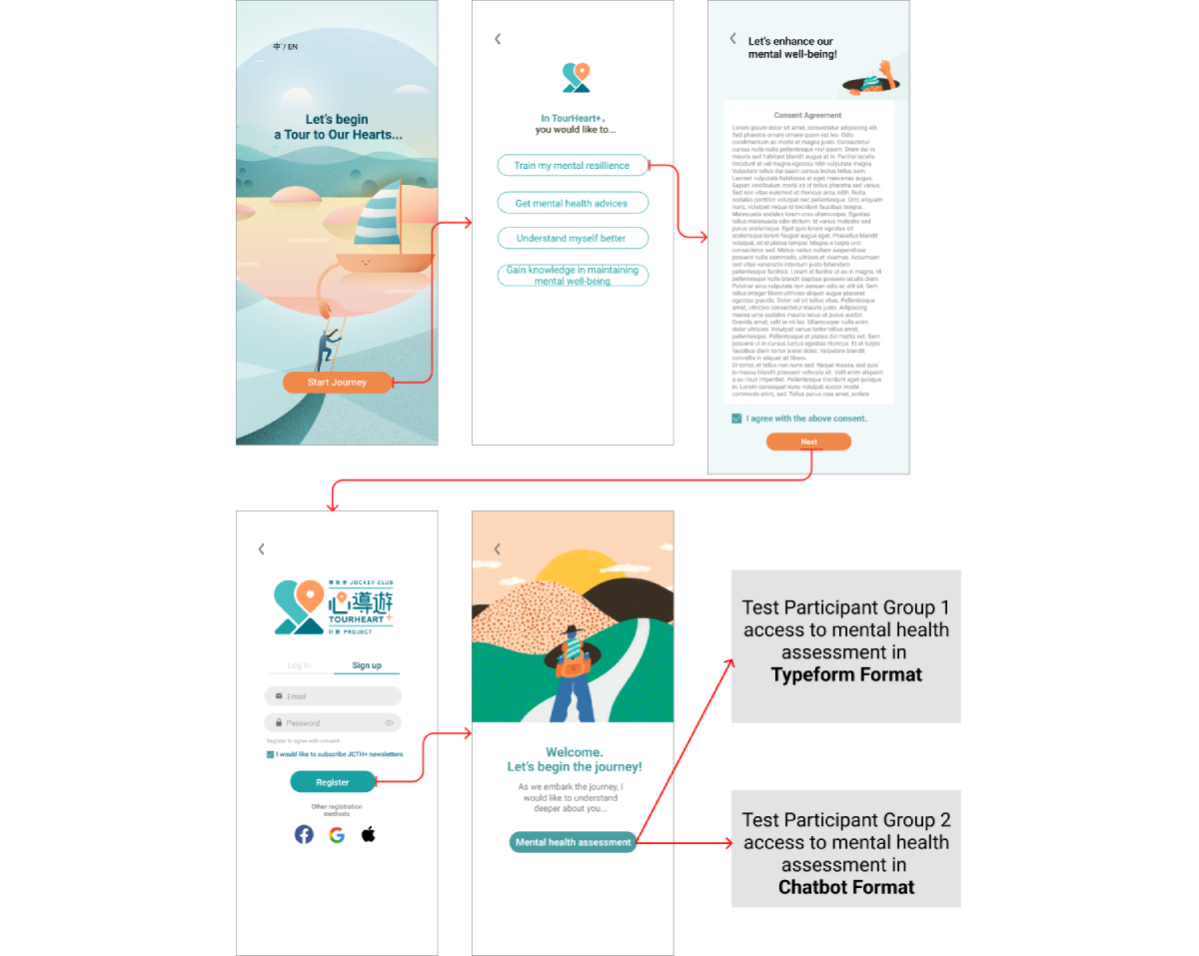
The flow of the prototype from account registration to mental health assessment in typeform or chatbot format.

The usability testing was conducted in August 2021. Participants received a link of the prototype and survey through email. They were randomly assigned to 2 groups, namely “typeform format prototype” and “chatbot format prototype” (Figure 3). The first section of the prototype consists of the registration flow, which includes a cover page of the platform, goal setting page, consent form for the platform, and account creation page. The second section brings participants to complete a mental health assessment in either “typeform format” or “chatbot format” according to their randomly assigned allocation.

#### Usability Testing on the Web-Based Self-help Courses

Usability testing on the courses and exercises of the platform was held through remote testing and the “think aloud” method that allows immediate comments from the users [39]. Participants were asked to use the prototype and simultaneously verbalize their thoughts. This formative testing allowed researchers to discover areas for improvement for the initial design of the courses and exercises to facilitate users’ learning and adherence. The contents of the self-help courses are based on internet-based mindfulness-based intervention [31,32] and internet-based rumination-focused cognitive behavioral therapy [40] that have been developed and evaluated previously and are intended for users with mild to moderate levels of anxiety and depressive symptoms.

**Figure 4:**
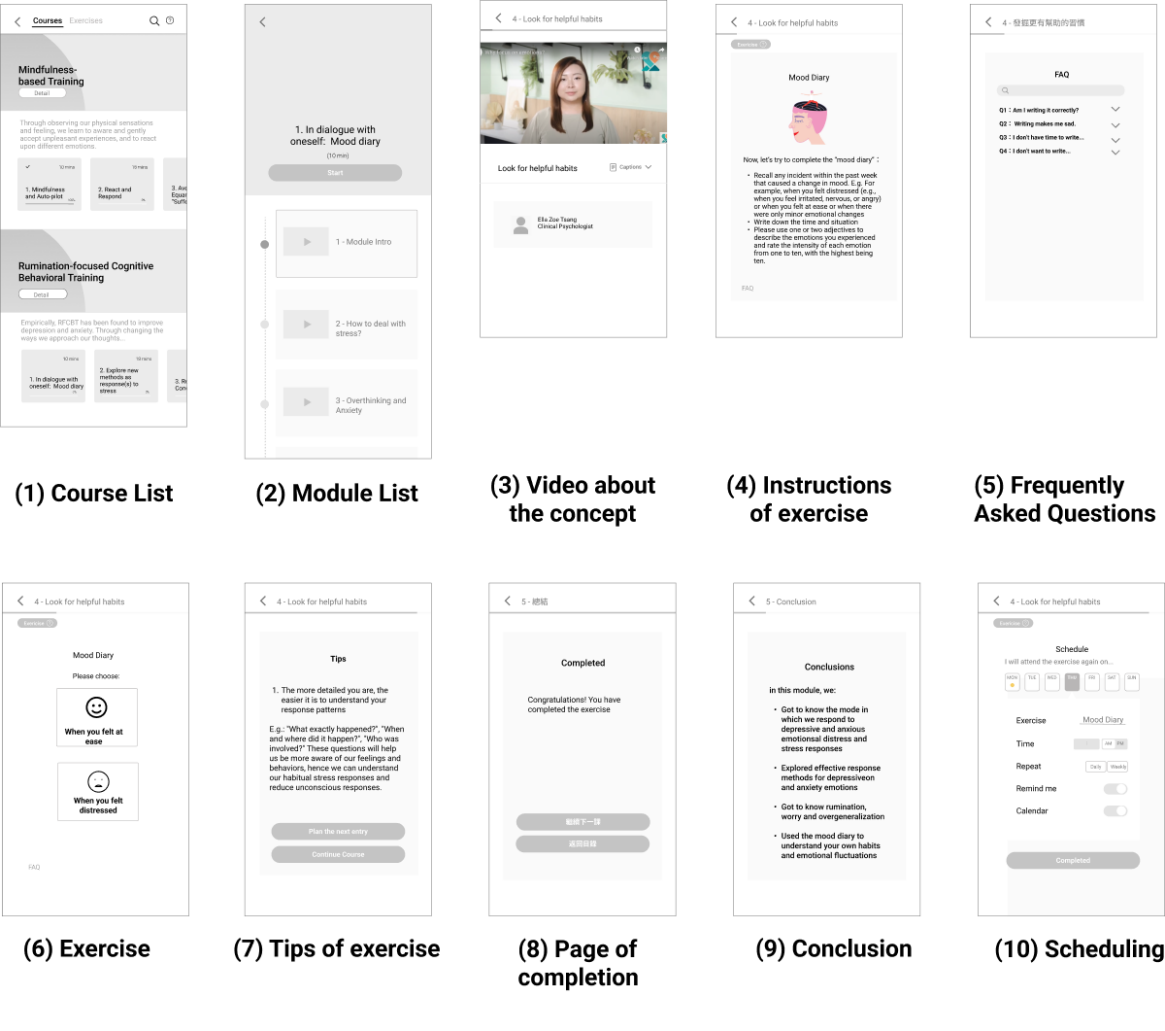
Flow of the prototype for usability testing on the web-based self-help courses.

The usability testing was conducted on July 6, 2021. Participants were asked to go through the prototype as a user intended to learn psychological methods for mental health self-care. With this goal in mind, participants went through the following steps (Figure 4): (1) course, (2) module list, (3) video about the concepts in a module, (4) instructions of the exercise, (5) frequently asked questions, (6) exercise, (7) tips of the exercise, (8) page of completion, (9) conclusion, and (10) scheduling. Upon completing all these steps, the UX researcher asked each participant a few open-ended questions.

#### Participants

Participants of the interviews and usability testing sessions were recruited through social media, mass emails to the authors’ university, and existing users of the TourHeart platform. The study targeted any potential users including previous users of TourHeart and those who have never used or heard of TourHeart.

For user interviews, interested individuals were required to complete a screening survey that consists of basic demographics (eg, age, gender, employment status, and occupation) and assessment of severity of the anxiety and depressive symptoms as measured by Generalized Anxiety Disorder-7 and Patient Health Questionnaire-9, respectively [41,42]. Given that TourHeart+ targets adults over a spectrum of mental wellness and common mental illness concerns, participants who are either in the work force or available for work are selected by the research team to ensure a relatively similar number of individuals across gender, age range, occupational status, and anxiety and depression levels. We aimed to recruit a minimum of 15 participants, with at least 5 participants having minimal, mild to moderate, or severe levels of distress.

For the 2 usability tests, participants were recruited using the same channels as user interviews on a rolling basis until the intended sample size was reached. For the usability test for the onboarding flow and mental health assessment, the target sample size was 100 participants, with 50 participants randomly assigned to the typeform condition and another 50 participants assigned to the chatbot condition, so as to reach sufficient power for statistical significance for this quantitative usability test. In the usability test for self-guided courses, a qualitative approach was taken. The target sample size was 5 participants because according to industry practices, a sample size of 5 is considered enough to uncover 85% of the issues under investigation [43].

#### Incentives

Upon completion of the study, each participant for the user interview received HK $100 (approximately US $13) and HK $50 (approximately US $6) for the usability tests.

### Ethics Approval and Informed Consent

This study obtained ethics approval from the Survey and Behavioral Research Ethics Committee of the Chinese University of Hong Kong (reference number: 6905754). All participants indicated their informed consent before the commencement of the study.

### Data Analysis

The data collected from the design workshop and the 2 usability testing were analyzed using an inductive approach and summarized as descriptive statistics if appropriate [44,45]. For the usability testing of the onboarding flow, participants rated the extent to which they find the experience private, interactive, clear, useful, and reliable on a 7-point Likert scale, with higher scores indicating more favorable ratings. Furthermore, 2-tailed *t* tests were conducted to compare possible differences in ratings between the typeform and chatbot formats. The UX researcher analyzed the data by identifying commonalities and differences to draw summaries clustered around common themes. The summaries were then sent to the team for checking and comments to ensure accuracy [46]. The user interviews were recorded and transcribed verbatim.

In developing the personas, attitudes, behaviors, and needs for mental self-care of the research participants were considered. A thematic coding approach was used for synthesizing the interview data to identify the variables under attitudes, behaviors, and needs. Participants with the most similarities across these 3 components were clustered into groups. Eventually, 4 groups with distinct characteristics were created. As a persona has to be a believable representation of our target users, we provided each persona with a nickname, characteristics, and preferences based on the findings, and supported them with users’ actual quotes [47].

As to forming a self-care journey, interview participants were asked to explain their experiences in dealing with mental health challenges. The key decisions and actions they did throughout the experiences are briefly mapped into timelines. On the timeline, 4 to 5 labels indicating the stages they have gone through were added. The timelines were compared for commonalities. An overarching self-care journey with 4 stages was formed eventually. Under the 4 stages, drivers and barriers that facilitate and hinder participants to move to the next phase of the journey were listed and summarized into points [48]. Quotes were also extracted to support the observations.

## Results

### Phase 1: Define Design Considerations by Empathizing With Users

#### Design Workshop

A design workshop was conducted with 11 team members, including scholars and researchers from psychology, data scientist, UX researcher, administrator, psychological well-being officers, and well-being promotion officers. They came from a diverse range of backgrounds, including psychology, engineering, business administration, digital marketing, and journalism.

#### Empathy Maps

Team members were divided into 2 groups to map out how would users with normal to mild and moderate to severe distress levels think, feel, hear, say, do, and see in relation to their mental health and what their gains and pain points were correspondingly in using mental health services.

The teams’ perceived thoughts, feelings, motivations, desires, and needs of target users’ mental health self-care experience are summarized below by the UX researcher.

Their emotional distress mainly come from stress and they would rely on their willpower to overcome it. Many are not open to talk about this as are afraid to be seen as “weak” or “vulnerable.” Gaining understanding on mental health and learning to take care of oneself mentally could help alleviate their distress. However, with a busy lifestyle and misconceptions on mental health in the society, many users face obstacles in prioritizing mental health in their lives and build a habit for mental health self-care.

Users with normal to mild levels of distress

With a more severe levels of psychological distress, they may feel helpless, suffocating, and hopeless. While they may try to find resonance and feel less lonely by reading real-life stories in the media, living in a society with mental illness stigma brings them struggles and challenges in their daily living. They may seek professional help or try various self-help methods but the lack of motivation and accessibility to self-help tools may create barriers to recovery. By having hope, patience, self-knowledge, and understanding on mental health, they may be more empowered to self-care.

Users with moderate to severe levels of distress

#### Point-of-view Statements

On the basis of the empathy maps, the team created point-of-view statements for these 2 groups of target users to help them empathize with possible perceived needs of users with different levels of distress. Examples of statements for users with a normal to mild level of distress were as follows: “They need a way to reflect on their mental health status because that helps them to recognize the issue and look for solution timely,” and “They need to turn self-care into a habit or way of life because it takes time and efforts to maintain mental well-being (just like physical exercise).” For users with a moderate to severe level of distress, the team thinks that “They need ways to be motivated along the recovery journey because they may encounter a lot of challenges,” and “They need a way to empower themselves to feel that well-being is possible because self-care is part of recovery.”

To summarize, the team believes that throughout a mental health self-care journey, it is important for them to be able to be aware of and reflect on their mental health status. While going through mental health recovery, the motivations, hopes, and the ability to make self-care a habit are also crucial for maintaining better mental well-being. The UX researcher designed the next user interview with these aspects in mind to explore the current approach of mental health self-care of our actual target audiences and the possibilities of incorporating these aspects into the platform design.

#### User Interviews

##### Participants

Out of 68 respondents who completed the screening questionnaire, 23 covering a range of demographic characteristics and distress levels were invited to join the interview. A total of 19 participants (including 13/19, 68% women; mean age 28.74, SD 6.19 years; 13/19, 68% employed) completed the user interviews, with the remainder canceling or not attending the scheduled interview. Most of them (15/19, 78%) were recruited through social media. Details of their demographic characteristics are shown in Table 3.

**Table 3:** Participant demographic data (N=19).

Characteristics			Participants, n (%)
**Age range (years)**			
	20-25	6 (32)	
	26-30	6 (32)	
	31-35	4 (21)	
	36-42	3 (16)	
**Gender**			
	Women	13 (68)	
	Men	6 (32)	
**Distress level**			
	Minimal	8 (42)	
	Mild to moderate	7 (37)	
	Severe	4 (21)	
**Occupation**			
	Corporate employees	7 (37)	
	Teachers	3 (16)	
	Health care workers	3 (16)	
	Unemployed	3 (16)	
	Students	3 (16)	

##### Forming Personas

After the interviews were transcribed, researchers coded the transcripts and came up with a 3-component model consisting of attitudes, behaviors, and needs toward mental health self-care. The major attitudes distilled from the transcripts included being hopeless, motivated, skeptical, and committed. As to behaviors, the main themes included learning about mental health regularly, escaping from challenges, finding practical solutions, and self-reflection. As to needs, participants indicated resources to learn, professional guidance, ways to articulate feelings, encouragements, and be reminded about self-care. In addition to these 3 major components expressed by the participants, other themes revolved around self-awareness of their own mental health status and their conceptions about mental health.

**Figure 5:**
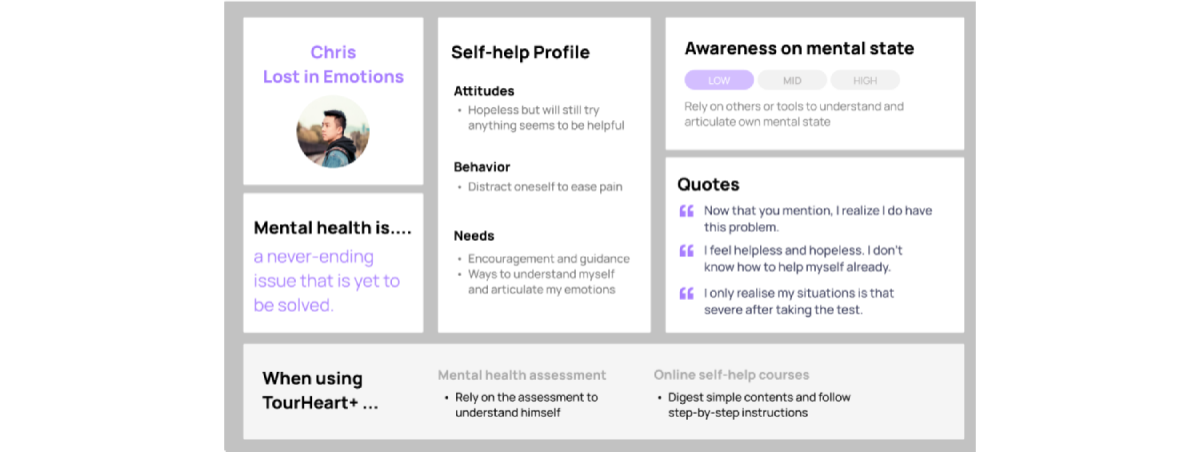
Persona of “Chris–Lost in Emotions”.

**Figure 6:**
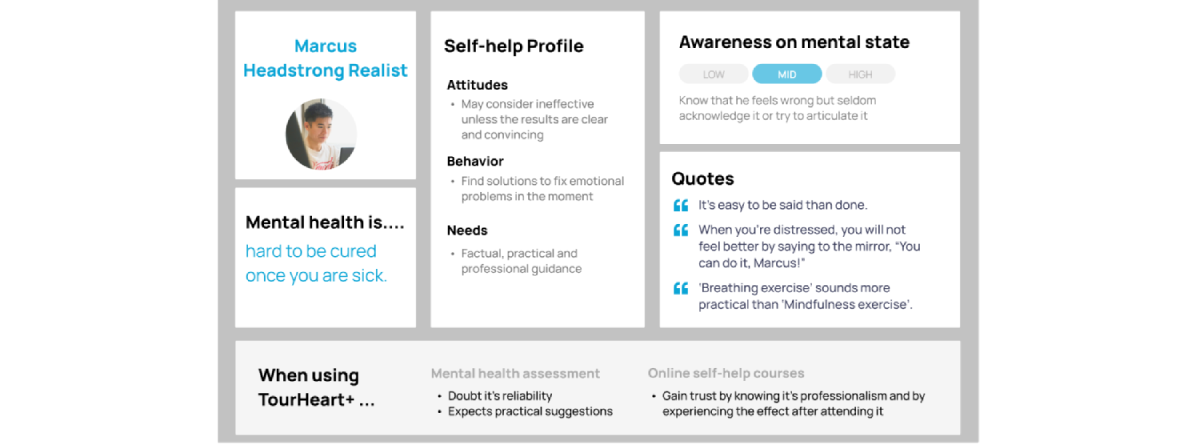
Persona of “Marcus–Headstrong Realist”.

**Figure 7:**
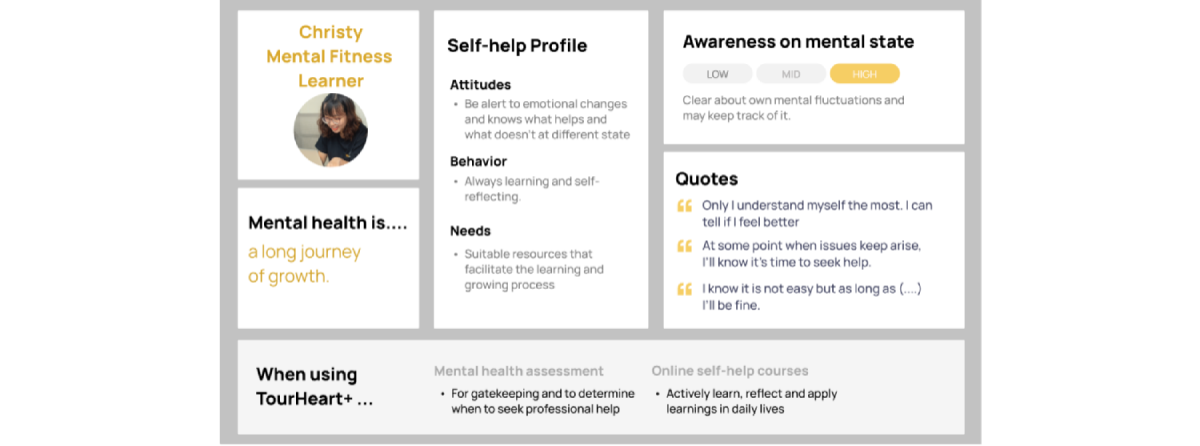
Persona of “Christy–Mental Fitness Learner”.

**Figure 8:**
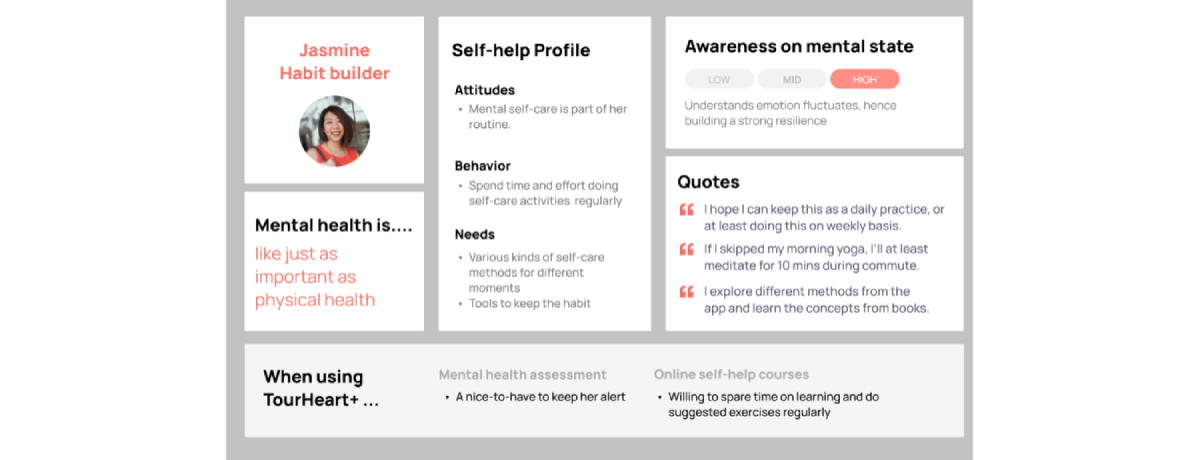
Persona of “Jasmine–Habit Builder”.

Clustering participants with similar characteristics along the above-mentioned components resulted in 4 personas. Figures 5-8 show the characteristics of the 5 personas, namely “Chris—Lost in Emotions” (Figure 5), “Marcus—Headstrong Realist” (Figure 6), “Christy—Mental Fitness Learner” (Figure 7), and “Jasmine—Habit Builder” (Figure 8). These personas were informative in making design decisions in relation to potential users having different user behaviors when using mental health assessment and web-based self-help courses.

##### Forming Self-care Journeys

User interviews also indicated that the mental health recovery journeys and self-care methods vary across individuals. As illustrated in Table 4, a total of 4 overarching steps were derived through the patterns drawn among the actions and decisions participants made throughout the experiences. Generally speaking, individuals experienced a period when they acknowledge changes in their mental health. Individuals also reported looking for ways to respond to the changes to obtain emotional and psychological relief. Participants mentioned learning to empower themselves to gain mental resilience and maintaining a self-care routine to support their mental well-being. It is important to note that these described journeys overlapped across personas and could be experienced nonlinearly instead of being necessarily sequential [49,50].

**Table 4:** The overarching steps of a self-care journey.

	Acknowledge	Respond	Empower	Adapt
Facilitators	Accept and reflect on mental health change	Able to find tools or methods for emotional relief at the moment	Actively learn to self-care Keep trying and finding suitable methods	Committed to making self-care a habit Stay alert with mental health changes
Barriers	Lack of self-awareness Unable to articulate emotions	Give up self-help when the mental health issue is not solved	Unable to find self-help resources Do not feel any positive changes	Not motivated to self-care when mentally stable

##### Potential Process During the Self-care Journey—Acknowledge

At this juncture, participants reported encountering issues in life that causes emotional distress. The issue could be related to work, romantic or family relationships, etc. They were able to recognize that they do not feel good. This realization leads some to seek help:

I did something wrong at work as a nurse and I felt really bad about it. I do not feel good every day when I go to work. This triggered me to think about myself.

Participant, 27 years, female, nurse

Earlier this year, I started to look back and realize I have not been happy in recent years. I have been lacking confidence and self-worth.

Participant, 26 years, male, teacher

It was not as bad but don't want to fall into the “abyss” of emotions again...[Participant, 42 years, female, corporate employee]

Some participants did not realize their distress and did not try to look for help to alleviate the distress:

You are right...now that when you show me the results, I realize I do worry I lot

[Participant, 39 years, female, corporate employee]

I didn’t realize how serious is my problem until then.

[Participant, 36 years, female, nurse]

##### Potential Process During the Self-care Journey—Respond

Participants reported having various responses toward distressing moments. The aim of these responses was mainly to gain immediate and momentary relief:

When I was very down and couldn’t even sleep, I would cry. I would talk with my husband or take Panadol to make myself sleep.

Participant, 42 years, female, corporate employee

I would mainly go for a walk or listen to music. I usually will stay away from home when I’m in distress.

Participant, 35 years, female, currently unemployed and looking for work

For some of the participants, what prevents them from feeling better is that they believe emotional distress is a problem that cannot be solved. They may give up looking for ways and merely allow the distress to stay or let them pass:

I thought I could only let time pass. I do not think there are remedies for that.

Participant, 27 years, female, nurse

I never try to face or to solve it and that’s why my worry snowballed...[Participant, 39 years, female, corporate employee]

##### Potential Process During the Self-care Journey—Empower

After several attempts of trying to alleviate their distress, some may start to be curious about self-care and try to look for ways to self-help. They may start searching on the web, reading books, and learning self-help methods to take care of their mental health:

...then I started to go online and search about it (mental health). I guess the more I read the related posts online, the more I become aware.

Participant, 27 years, female, nurse

I promised by friends that I would find them whenever I have panic attack. I would force myself not to hide my feelings. I would attend talks about emotional management at school as well. I have a logbook recording the moments I have panic attack too.[Participant, 26 years, male, teacher]

Some may give up as they could not feel any effect after trying various ways. A few may give up because they do not know any mental health self-help methods:

I believe I have tried everything I could...I am very frustrated. I do not know what else I could do.

Participant, 28 years, female, currently unemployed and looking for work

I am not so clear about the details. I only tried finding a good family counselor but in vain. Then I didn't continue exploring.[Participant, 27 years, male, corporate employee]

##### Potential Process During the Self-care Journey—Adapt

During adaptation, participants were able to find ways that are effective and are gradually making progress to attain better mental well-being. Many practiced self-care regularly to maintain their mental well-being:

Now, every morning I’ll do 20-min yoga plus 10-min meditation before making breakfast...If I am in a rush for work sometimes, I will still meditate on the bus. This is my bottom line of self-care.

Participant, 39 years, female, corporate employee

Participants who fail to adapt to a self-care routine tend to consider self-care as something to do only when feeling distressed:

I would do “worry time” exercise during stressful period. Now I feel healthier so I didn’t really do anything.

Participant, 26 years, male, teacher

I joined group therapy events before...(How about now? Are you still practicing what you have learnt?) Now, I am lazy to do so and I feel a lot less distressed after taking a long break.

Participant, 27 years, female, nurse

#### Design Considerations

Insights from the user interviews informed the development team of several design considerations based on the 4 personas and processes along the self-care journey. First, it is important to educate users about self-care with clear explanations, guidance, and practical exercises. With respect to mental health assessment, the results should provide clear and comprehensive explanation to help users understand their own mental health status. Users sharing similar concerns as the persona “Chris—Lost in emotions” find it challenging to articulate or notice their mental health changes. Users may rely on mental health assessment to understand themselves and gain self-awareness. Hence, mental health assessment needs to guide users to acknowledge their mental health change, which facilitates them to complete the first step of the self-care journey in Table 4.

In addition, the assessment results and contents of the web-based courses should be able to provide practical solutions or suggested actions. When distress arises, users who shared the persona “Marcus—Headstrong Realist,” tend to find practical solutions to “fix” their distress at the moment. If the effect is not immediate, some may give up trying. As such, practical suggestions and hands-on self-help exercises that are evidence-based or found to be effective could be included in the assessment results and in the course contents.

To motivate users to develop self-care as an ongoing practice, the platform also needs to proactively nudge users and make mental health resources accessible. This way, all users can access different mental health resources from the platform easily. Nudges can also help to enhance platform engagement and course adherence. Users who shared the persona “Jasmine—Habit builder,” consider mental health self-care as part of their daily routine. Participants who reported having better mental well-being tended to stay alert and be committed to make self-care a habit. However, some participants were not motivated to reach out to mental health resources unless they are experiencing mental health struggles. Other users who shared the persona “Marcus—Headstrong Realist,” tended to take care of their mental well-being only when it becomes a problem to them instead of keeping self-care an ongoing habit. To facilitate users in developing a habit for self-care, notification features and scheduling tools will be added to the platform design. Through the scheduling function, users will be reminded to complete a self-care exercise suggested in the course. This proactive way of reminding users can encourage users who may be less self-initiating or motivated to give the self-care exercises a try and potentially practice them as part of their daily routine to maintain their mental well-being.

An overall design consideration based on insights from these user interviews was the importance of having the platform exude a sense of trust, reliability, and professionalism to potential users with different mental health-related needs, attitudes, and behaviors. The development team was reminded to inform the users that the contents are evidence-based and were designed by experts with a clinical psychology professional background. Especially users who shared the persona “Marcus- Headstrong Realist,” tended to be skeptical about the effects of self-care because they perceived it as lacking professional involvement. To increase understanding of the rationale of self-care, the development team will explicitly state the purpose and background of the mental health assessment as well as related references and evidence base that supports the web-based courses. The involvement of clinical psychologists in the development of these web-based courses and their related credentials are provided on the front page of the web-based courses to increase the courses’ credibility.

### Phase 2: Improve Platform Usability by Testing Prototypes With Target Users

#### Usability Testing on Onboarding Flow Including Registration and Mental Health Assessment

##### Participants

A total of 104 individuals participated in this usability test. Among the 104 participants, 75% (78/104) were women and 75% (78/104) were employed; they were aged between 18 and 30 years (50/104, 49%), 31 and 40 years (30/104, 28.8%), 41 and 49 years (9/104, 8.7%), and ≥50 years (13/104, 13%). Their mean depression (Patient Health Questionnaire–9) score was 7.85 (SD 5.16), and anxiety (Generalized Anxiety Disorder-7) score was 6.99 (SD 4.72). Details of their demographic characteristics are shown in Table 5.

**Table 5:** Participant demographic data.

Characteristics			Typeform format prototype (n=56), n (%)		Chatbot format prototype (n=48), n (%)
**Age (years)**					
	15-20	1 (2)		0 (0)	
	20-25	10 (18)		21 (44)	
	26-30	14 (25)		9 (19)	
	31-35	12 (21)		4 (8)	
	36-40	5 (9)		5 (10)	
	41-49	4 (7)		4 (8)	
	50 or above	9 (16)		0 (0)	
**Gender**					
	Women	40 (72)		38 (79)	
	Men	16 (29)		10 (21)	
**Distress level**					
	Minimal	17 (18)		10 (21)	
	Mild to moderate	38 (68)		38 (79)	
	Severe	1 (13)		0 (0)	
**Occupation**					
	Corporate employees	38 (68)		39 (80)	
	Teachers	6 (11)		3 (6)	
	Health care workers	4 (7)		0 (0)	
	Unemployed	5 (9)		2 (4)	
	Students	3 (5)		4 (8)	

##### Results of Usability Testing on Registration and Mental Health Assessment

For the usability testing on the onboarding flow of the registration and mental health assessment, although ratings for the chatbot condition were consistently higher than the typeform condition, they were not statistically different (Table 6). In other words, participants found the instructions and assessment results to be clear, the assessment process interactive, and the mental health assessment results to be reliable and useful. Participants between both conditions also indicated interest in using the platform, with the platform generally being considered as private. In both typeform and chatbot conditions, 69.2% (72/104) of the participants found the onboarding flow friendly and 49% (51/104) were feeling cared for with no considerable difference between the 2 formats. Comparing the 2 conditions, a higher percentage of participants in the chatbot condition (14/104, 13.5%) reported finding the onboarding flow interesting, compared with the typeform condition (8/104, 7.7%).

Overall between both conditions, participants’ perceived functions and purposes of using TourHeart+ aligned with the needs of the 4 personas. Specifically, upon experiencing the onboarding flow with registration and mental health assessment, most of the participants had the impression that the platform is for self-understanding through check-ups, privately seeking help to address emotional needs, gaining mental health knowledge, and maintaining and improving one’s mental well-being. These perceived functions matched with the needs expressed during the target users’ interviews in which participants wanted to find ways to understand themselves and articulate their emotions, have resources that were suitable for their personal growth and learning for mental health improvement, and obtain practical and professional guidance for mental health self-care. Table 7 describes user feedback on each section of the prototype, “welcome screens and registration,” “chatbot,” “mental health assessment,” and the corresponding design implications.

**Table 6:** Quantitative results of the usability testing on registration and mental health assessment and independent sample *t* test results comparing typeform and chatbot on various aspects of usability^a^.

Usability metrics	Mental health assessment format, mean (SD)			*t* test (*df*)		Significance (2-tailed), *P* value
	Typeform (n=56)	Chatbot (n=48)				
Interested in using the platform	4.91 (1.20)	5.17 (1.10)	−1.432 (106)		.16	
Privacy of using the platform	4.59 (1.49)	4.77 (1.46)	−0.727 (106)		.47	
Clarity of instructions	5.18 (1.44)	5.44 (1.20)	−1.304 (106)		.20	
Interactivity of mental health assessment	4.04 (1.15)	4.24 (1.10)	−1.038 (84)		.30	
Clarity of mental health assessment result	4.38 (1.13)	4.53 (1.13)	−0.571 (85)		.57	
Reliability of mental health assessment result	4.70 (1.16)	4.71 (1.07)	−0.421 (106)		.68	
Usefulness of mental health assessment result	4.63 (0.95)	4.83 (0.93)	−1.362 (106)		.18	

^a^Ratings ranged from 1 to 7.

**Table 7:** Pain points and opportunities to improve the platform flow.

	User feedback	Implications
Welcome screens and registration	Like the visual designs and find the quotes meaningful	Keep using and make good use of the graphics in various pages
Chatbot	Interactive and caring as if talking to a real person A few find chatbot mechanical or nonhuman like	Though the attitudes toward chatbot may vary among users, the interactive and caring impression could be further amplified in chatbot dialogue design
Mental health assessment	Perceived the 16 items of PHQ^a^-9 and GAD^b^-7 being too basic to identify one’s mental state, hence may not be too reliable or useful Some find that the assessment is too long Perceived it as professional but some expects to see references to increase its reliability Convenient and efficient to complete the assessment	More information and references about PHQ and GAD could be added in the dialogue to enhance professionalism and credibility The importance and purpose of taking the assessment could also be highlighted before the assessment to motivate users to complete the 16 questions

^a^PHQ: Patient Health Questionnaire.

^b^GAD: Generalized Anxiety Disorder.

#### Usability Testing on Web-Based Courses

##### Participants

The 5 participants [43] (2/5, 40% women; 3/5, 60% men) aged between 20 and 29 years (3/5, 60%) and 30 to 39 years (2/5, 40%), with 60% (3/5) being corporate employees at different management levels, 20% (1/5) being unemployed, and 20% (1/5) being a student.

##### Results of Usability Testing on Web-Based Courses

**Figure 9:**
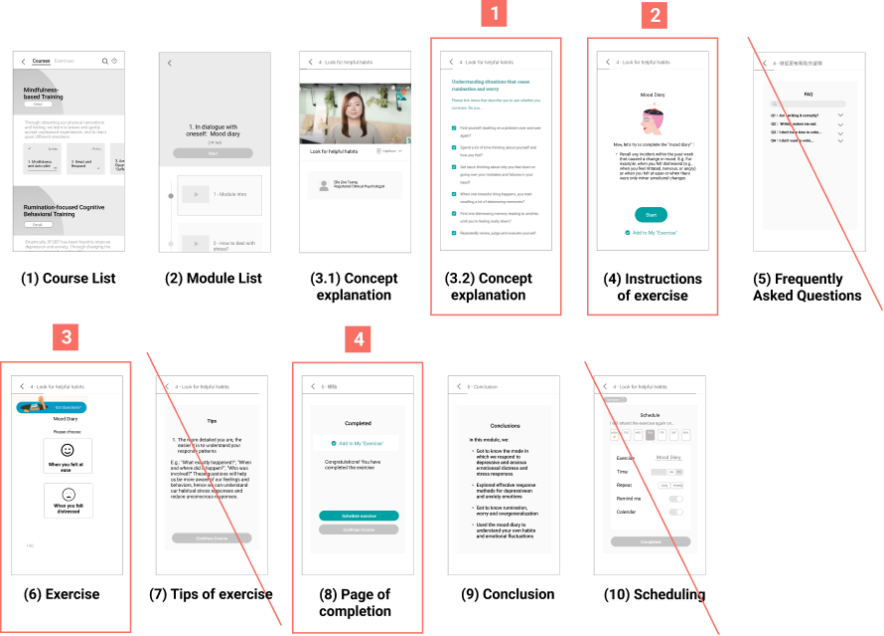
Original flow for a course and exercise with amendments based on the insight.

During usability testing, it is observed that participants were unable to recall the key messages of the sessions or instructions of the exercise. They were overwhelmed by all the contents presented within 1 session. Given that self-care and many cognitive behavioral exercises were novel to the participants, they needed to revisit the pages and read the contents repeatedly. These user behaviors shed light on the importance of breaking down the content into simpler concepts and highlighting it in key points or questions-and-answers to facilitate understanding as shown on point 1 in Figure 9. The courses also need to be easily navigated back and forth and the learned exercises should be easily accessible, rather than being embedded within the sessions, to ease retrieval. First, the design of the web-based courses can be improved by having clear demarcation of the start and end of an exercise section so that users can go back later for practice without going through the course contents again. As displayed in point 4 of Figure 9, “Add to Exercise” was added to indicate that exercises being visited were automatically added to an exercise library for users to access. Second, the course design should maximize the autonomy of users to start, skip, and schedule any exercises based on their readiness to practice and preferred time to ease their navigation of the content. Points 2 and 4 in Figure 9 show the buttons suggested to be added on the start and end page of an exercise. Finally, tips and frequently asked questions can be grouped and accessed on every page throughout the exercise through a “Got question?” button, as shown on point 3 in Figure 9. These changes in the interface design shall enhance understanding of the course materials and offer guidance in completing the exercise.

## Discussion

### Principal Findings

This study described the iterative design process in developing TourHeart+, a web-based platform using a stratified stepped care approach for mental well-being, through user-centered design. This early design phase of development included a design workshop with the development team, users’ interviews, and 2 usability testing sessions for registration flow including mental health assessment and the web-based courses. In this study, the team’s perceptions of users’ needs and expectations were first gathered, then they were verified and refined from users’ interviews that tapped into users’ perceptions, attitudes, and behaviors toward self-care. This first phase of the study orientated the development team to empathize with target users on their mental health needs and self-care practices and consider platform design and functions from the users’ point of view. This process is essential in developing a user-friendly and responsive platform that was tested in subsequent usability testing sessions in phase 2 to further refine the design to enhance technology adoption [51].

Users’ interviews explored real-life experiences of target users and enabled the team to relate users’ real-life experiences with the contents of the platform. Consistent with past studies [52-61], findings showed that users have different personas differing in their attitudes, behaviors, and needs toward self-care. Facilitators and barriers were mapped on each step of the journey of self-care. The persona and the journey were informative in making design decisions as they give a representation of our potential users regarding who they are and how they are practicing self-care.

The users’ personas and mental health self-care journey summarized how users with different profiles may approach self-care and mental health. Findings indicated the importance for users to be aware of and reflect on their mental health status to motivate them to practice self-care. This observation is consistent with previous studies showing that individuals need to be mindful of their self-care to promote practice and well-being [62,63]. This awareness interacts with users’ motivations, hopes, and ability to make self-care a habit, which results in different preferences and approaches to maintain mental well-being. This empathy process is essential in all user-centered mental health platform design to sharpen the platform’s abilities to address target users’ mental health needs and concerns. Specifically, phase 1 culminated into several points to be heeded to in the design of the platform. First, it is important to guide and educate users in being aware and acknowledging their mental health change through the mental health assessment. Second, practical suggestions with hands-on exercises are to be displayed as an attempt to let users experience the immediate effect. Third, to facilitate users to develop self-care habits, it is crucial to add features to motivate and remind users, including adding notifications or scheduling tools. Fourth, it is important to exude a sense of trust, reliability, and professionalism by informing users that the contents are evidence-based and are designed by clinical psychology professionals. Phase 2 consisted of 2 usability testing sessions on the registration flow, mental health assessment, and web-based courses, which are the core contents of the platform. Findings collected from the usability testing sessions informed the team on ways to enhance platform functions in the subsequent iterative development process.

In summary, it is crucial for designers to understand the experiences and behaviors of users in relation to self-care to make relevant design decisions. Initially, designers can segment their audience based on distress levels and demographics, and design based on their assumptions. However, it is important to conduct continuous user research to gain a deeper understanding of users’ attitudes, behaviors, needs, and current experiences. This includes conducting user interviews, usability testing, and other forms of research. Through this process, designers can better empathize with the users and design a platform that accounts for users’ various profiles and needs, supports self-care, and offer user-friendly and evidence-based interventions on the internet for the target audience.

### Limitations

Several limitations of this study warrant caution. This study presents findings reflecting perspectives from adults in Hong Kong, with most of the users being Hong Kong Chinese women. Thus, findings may not be generalizable to other populations. Further research with both quantitative and qualitative design should be conducted to substantiate the findings of the study with diverse populations. With the exception of the survey, the sample size for user testing sessions and usability test was small. Self-report might also be subject to social desirability bias and subjective bias. Nevertheless, the methods used in this study are appropriate for user-centered design during the formative stage of platform development.

### Conclusions

The iterative process and findings presented in this study are important for developing a web-based stratified stepped care mental health platform based on a user-centered design that optimizes users’ usability and acceptance. The TourHeart+ platform has the potential in providing a variety of evidence-based mental health care options for people to access self-care and psychological interventions at any time, any place, which cannot be met through existing mental health services in the community. This design process is essential in integrating scientific evidence, potential users’ real-life concerns and needs, and users’ experiences in platform development.

## Abbreviations

UX: user experience
